# Natural language processing (NLP) to facilitate abstract review in medical research: the application of BioBERT to exploring the 20-year use of NLP in medical research

**DOI:** 10.1186/s13643-024-02470-y

**Published:** 2024-04-15

**Authors:** Safoora Masoumi, Hossein Amirkhani, Najmeh Sadeghian, Saeid Shahraz

**Affiliations:** 1 Pediatric Infectious Diseases Research Center, Mazandaran University of Medical Sciences, Sari, Iran; 2 Computer and Information Technology Department, University of Qom, Qom, Iran; 3 Student Research Committee, Mazandaran University of Medical Sciences, Sari, Iran; 4 Institute for Clinical Research and Health Policy Studies, Tufts Medical Center, Boston, USA

**Keywords:** Natural language processing (NLP), BioBERT, Trend analysis, Machine learning, Medicine

## Abstract

**Background:**

Abstract review is a time and labor-consuming step in the systematic and scoping literature review in medicine. Text mining methods, typically natural language processing (NLP), may efficiently replace manual abstract screening. This study applies NLP to a deliberately selected literature review problem, the trend of using NLP in medical research, to demonstrate the performance of this automated abstract review model.

**Methods:**

Scanning PubMed, Embase, PsycINFO, and CINAHL databases, we identified 22,294 with a final selection of 12,817 English abstracts published between 2000 and 2021. We invented a manual classification of medical fields, three variables, i.e., the context of use (COU), text source (TS), and primary research field (PRF). A training dataset was developed after reviewing 485 abstracts. We used a language model called Bidirectional Encoder Representations from Transformers to classify the abstracts. To evaluate the performance of the trained models, we report a micro f1-score and accuracy.

**Results:**

The trained models’ micro f1-score for classifying abstracts, into three variables were 77.35% for COU, 76.24% for TS, and 85.64% for PRF.

The average annual growth rate (AAGR) of the publications was 20.99% between 2000 and 2020 (72.01 articles (95% *CI*: 56.80–78.30) yearly increase), with 81.76% of the abstracts published between 2010 and 2020. Studies on neoplasms constituted 27.66% of the entire corpus with an AAGR of 42.41%, followed by studies on mental conditions (*AAGR* = 39.28%). While electronic health or medical records comprised the highest proportion of text sources (57.12%), omics databases had the highest growth among all text sources with an AAGR of 65.08%. The most common NLP application was clinical decision support (25.45%).

**Conclusions:**

BioBERT showed an acceptable performance in the abstract review. If future research shows the high performance of this language model, it can reliably replace manual abstract reviews.

**Supplementary Information:**

The online version contains supplementary material available at 10.1186/s13643-024-02470-y.

## Background

The history of natural language processing (NLP) is relatively short, but it has seen rapid growth through multiple fundamental revolutions. Alan Turing invented a test in the 1950s to determine whether computers could think like humans [1]. NLP scientists then applied universal linguistic rules to textual data to understand it. During this time, Noam Chomsky’s universal theory of language dominated NLP scientists’ attention. Computer scientists replaced this linguistic approach with computational models based on statistical analysis [1]. Increasing computational power for analyzing a large amount of textual information has contributed to our current understanding of NLP and its applications due to the invention of machine learning methods, especially deep learning [1–3]. Our intelligent machines now need natural language processing (NLP) to decipher meanings from human languages. With the widespread availability of smart gadgets in everyone’s life, NLP has become even more advanced over the past two decades [4, 5]. Machines cannot recognize phrases and expressions without NLP in spoken and written languages. Moreover, the enormous amount of unstructured data produced daily highlights the need for NLP to assist professionals in sorting out their information [2, 4]. Evidence-based medicine relies on systematic literature reviews to answer specific questions from a large amount of textual data, which can be challenging and time-consuming [6].

Machine learning and natural language processing can speed up and improve the SLR. In this context, text classification and data extraction are two NLP-based strategies. Abstract screening is an essential application of text classification in literature reviews. Alternatively, data extraction identifies information about a particular variable of interest. NLP can, for example, help extract the number of individuals who participated in particular clinical trials [6, 7]. BERT (Bidirectional Encoder Representations from Transformers) is a transformer-based machine learning model for language modeling that has demonstrated significant success in various NLP tasks [8]. BioBERT, a BERT-based model pre-trained on biomedical texts, has outperformed other pre-trained language models in some biomedical datasets [9]. BioBERT has been highly performant in previous studies [10–13].

In this study, we deliberately analyze the evolution of medical NLP over the last two decades and benchmarked some of our findings against two similar studies published recently [14, 15]. As an example of how NLP aids abstract review, we conducted an SLR using an automated method. Based on the results of SLR, a list of data sources used in medical NLP literature is provided, along with the type of NLP application and the related disease areas. We also show how the BioBERT model categorizes abstracts.

## Methods

### Developing training data

PubMed, Embase, PsycINFO, and CINAHL were searched using controlled vocabulary thesaurus (MesH in PubMed and Emtree in Embase) and free-text keywords. The search queries included “natural language processing” and “text mining.” Additional file 1 provides the full search queries. Also excluded were editorials, case reports, commentary, erratum, replies, and studies without abstracts. Before 2000, there were few NLP studies. Therefore, we included all abstracts published between January 2000 and December 2020. Multiple steps are involved in the study. First, we classified NLP abstracts based on their text source (e.g., social media versus clinical notes). After optimizing retrievable meaningful classes of abstracts, a finalized training dataset was created. Next, we calculated the classification accuracy of the computer algorithm using the entire corpus. As a final step, we applied the algorithm to obtain the classes and visualized them. The last author (S. S.) randomly selected 100 abstracts from PubMed and classified their text sources, the context of use (e.g., abstracts pertaining to clinical decision support vs. those related to NLP method development), and the type of medical conditions studied. Using these primary classes, the lead author (S. M.) and third author (N. S.) explored more classes and categories for each of these classes in further PubMed abstracts. By adding more abstracts, they continued to find more classes and subgroups until they were unable to find any more classes and subgroups. The saturation process was completed after reviewing 485 abstracts. All authors discussed and optimized the classification iteratively until they reached an agreement on the final classification. In Table 1, the finalized classes and their definitions are described.

**Table 1:** Definitions used in classifying the NLP abstracts

***Context of Use:***
** Clinical decision support and similar fields**
Studies involved NLP used in diagnosis, prognosis, treatment, outcome, or epidemiological information of a disease (e.g., trend analysis)
** NLP method advancement**
NLP studies with a new NLP method introduced
** Other medical fields**
Medical studies, not categorized in ICD-11 as a disease. These studies include complementary medicine, radiation oncology, nuclear medicine, physician-patient relations, drug research and development, patient-physician communication, chronic diseases, radiology, dentistry, research, pharmacovigilance, bacterial culture, and antimicrobial susceptibility reports, pharmacology, trend analysis, health education, vaccination, vaccine research, and clinical term normalization
** Bioinformatics**
NLP used in bioinformatics
** Waste basket collections**
Articles not related to medicine
***Text Source:***
** Not related**
Studies not related to medicine
Medical studies not associated with NLP
** Electronic medical/health and similar databases**
Studies when NLP ran on Electronic Medical/Health Records or similar databases
** Published medical evidence**
Studies with NLP ran on published medical texts (e.g., published peer-reviewed articles)
** Interview**
Studies with the source being data extracted from interviews (e.g., patient interviews)
** Not defined**
The text source was not identifiable
** Questionnaire**
Data extracted from questionnaires
** Social media**
Social media used as a source
** Website**
Website data such as Wikipedia as a source
** Omics databases**
Source being data extracted from omics repositories
***Primary Research Field:***
All medical conditions indexed in ICD-11 22-item chapters

*NLP* ; natural language processing, *ICD* ; International Classification of Diseases

### Analysis

**Fig. 1:**
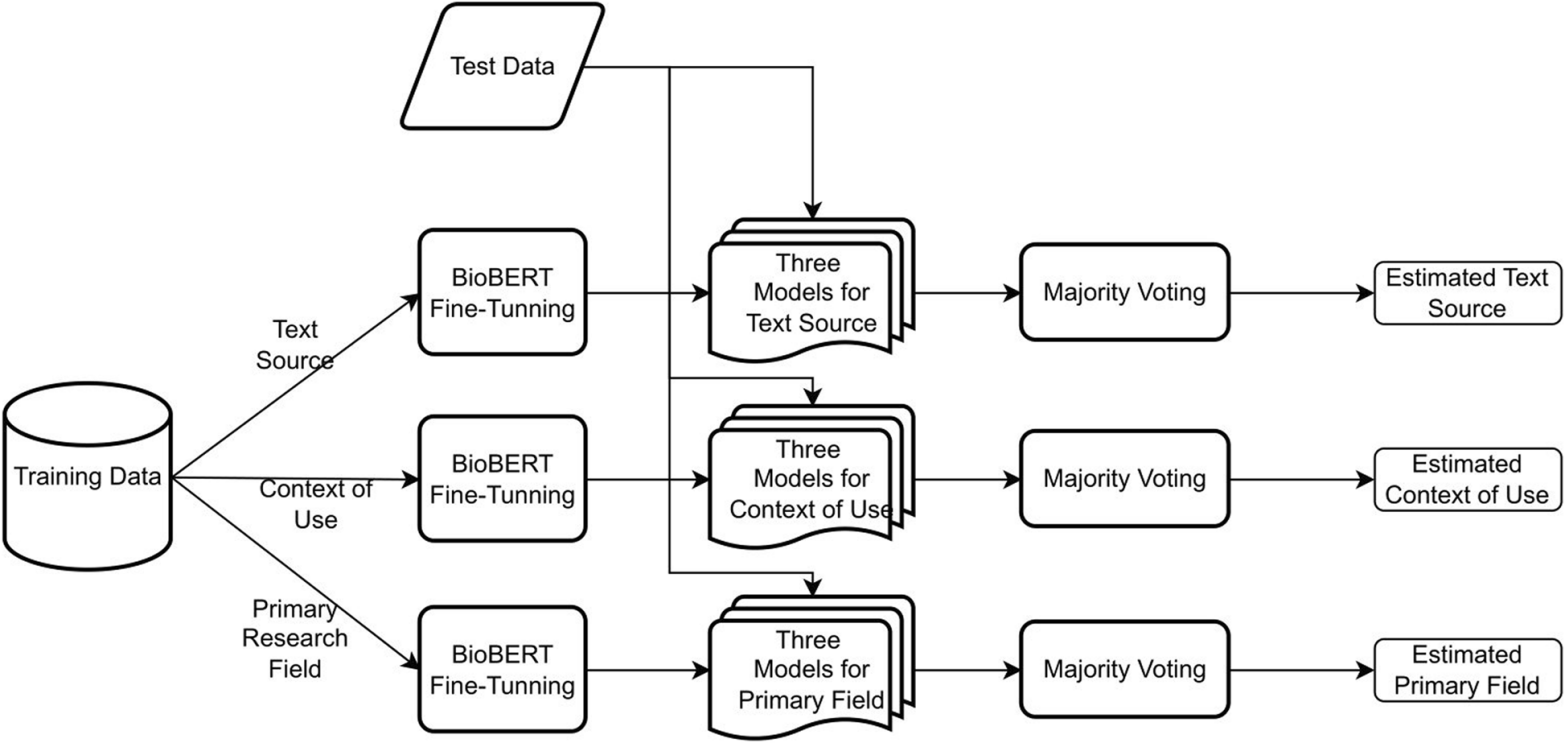
Overview of the proposed machine learning approach

As depicted in Fig. 1, machine learning algorithms were used to classify abstracts in the final corpus into those obtained from the trained dataset. By fine-tuning the pre-trained language models ubiquitous in modern NLP, we followed the favored approach. BERT, or Bidirectional Encoder Representations from Transformers, is a language model developed by Google [8]. The models are pre-trained on large corpora and then fine-tuned using task-specific training data by reusing the parameters from the pre-trained models. We used the BioBERT model [9] from the Hugging Face Transformers library [16], which was trained on abstracts from PubMed and full articles from PubMed Central. Then we fine-tuned three different models, one for each of our targets: text source, context of use, and primary research fields. The hyper-parameters, such as the learning rate and number of epochs, were selected using cross-validation. The final model was trained on the entire training data using the optimized hyperparameters. Since we utilized a pre-trained BioBERT model, a standard GPU, such as the Nvidia Tesla K80, was sufficient for fine-tuning the model during both the training and inference phases. All the experiments were conducted in a Google Colab environment leveraging this GPU.

For each target variable, we fine-tuned three different classifiers as an alternative method of improving the models’ accuracy. Repeating the fine-tuning process resulted in a different classifier due to different data batches used during training. The final prediction for an input article was obtained by majority voting of the base classifiers’ predictions. Afterwards, the trained models were applied to the entire corpus. A set of 362 randomly selected abstracts was manually annotated by the lead (S. M.), third (N. S.), and last author (S. S.) to evaluate the labels provided by the trained models. Next, the human annotations were compared to those provided by the models. The evaluation showed that the trained models’ accuracy in classifying abstracts into the text source, the context of use, and the primary research field was sufficient, mainly to track the time trends of the classes. Therefore, we assumed that misclassifications would remain constant over time. Our next step was to fit models that indicated publication growth rates for different study subgroups using ordinary least-squares regression. Citations were the dependent variable, and publication year was the predictor. Per year, the coefficient of the predictor showed an average increase in citations. A squared term for the publication year was added to the primary model to determine if the growth was linear or exponential. The increase in *R*^2^ indicated logarithmic growth. The average annual growth rate (AAGR) was calculated by averaging all annual growth rates (AGR) over the study period (sum of AGRs/number of periods). We calculated AGR as the difference between the current year’s value and the past year’s value divided by the past year’s value.

We report a micro f1-score to evaluate the trained models. The f1-score is calculated as the harmonic mean of precision and recall for the positive class in a binary classification problem.

True positive (TP) and true negative (TN) are the numbers of samples correctly assigned to the positive and negative classes, respectively. On the other hand, false positive (FP) and false negative (FN) are the numbers of samples that are wrongly assigned to the positive and negative classes, respectively. Accuracy is the ratio of the samples correctly assigned to their respective classes.

Precision (P) and recall (R) are calculated as follows if TP, FP, and FN represent the number of true-positive, false-positive, and false-negative instances, respectively:

$$P=\frac{TP}{TP+FP}$$

$$R=\frac{TP}{TP+FN}$$

And f1-score will be as follows.

$${f}_{1}=\frac{2PR}{P+R}$$

The average of the f1-scores obtained for different classes is computed for multiclass problems, such as ours. We report the weighted average considering the number of instances in each class in order to account for label imbalance.

## Results

**Fig. 2:**
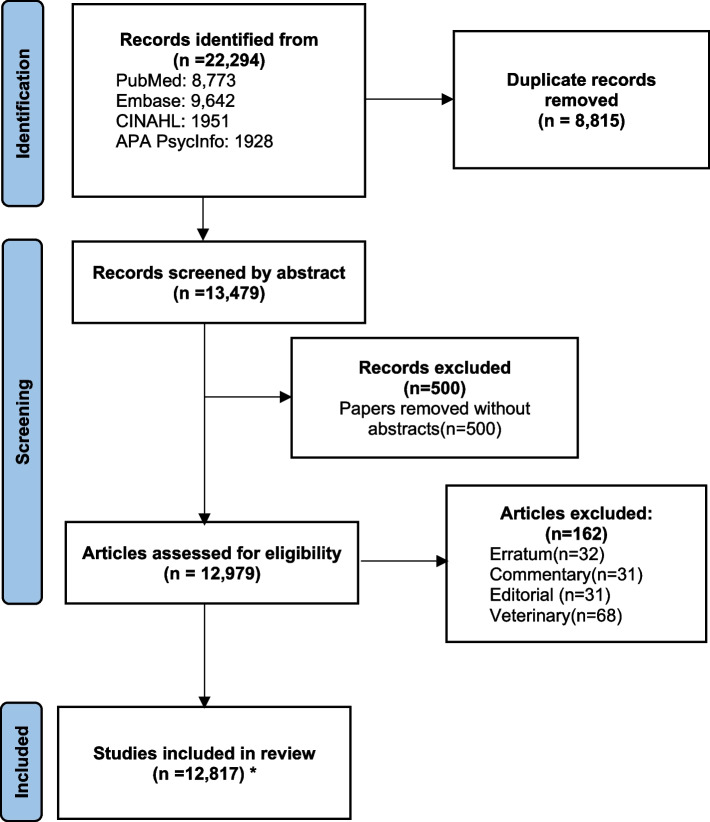
PRISMA flowchart illustrating the steps of abstract selection for building the final corpus. *For most analyses, we excluded abstracts for the year 2021, leaving 12,161 abstracts in the analysis data

**Fig. 3:**
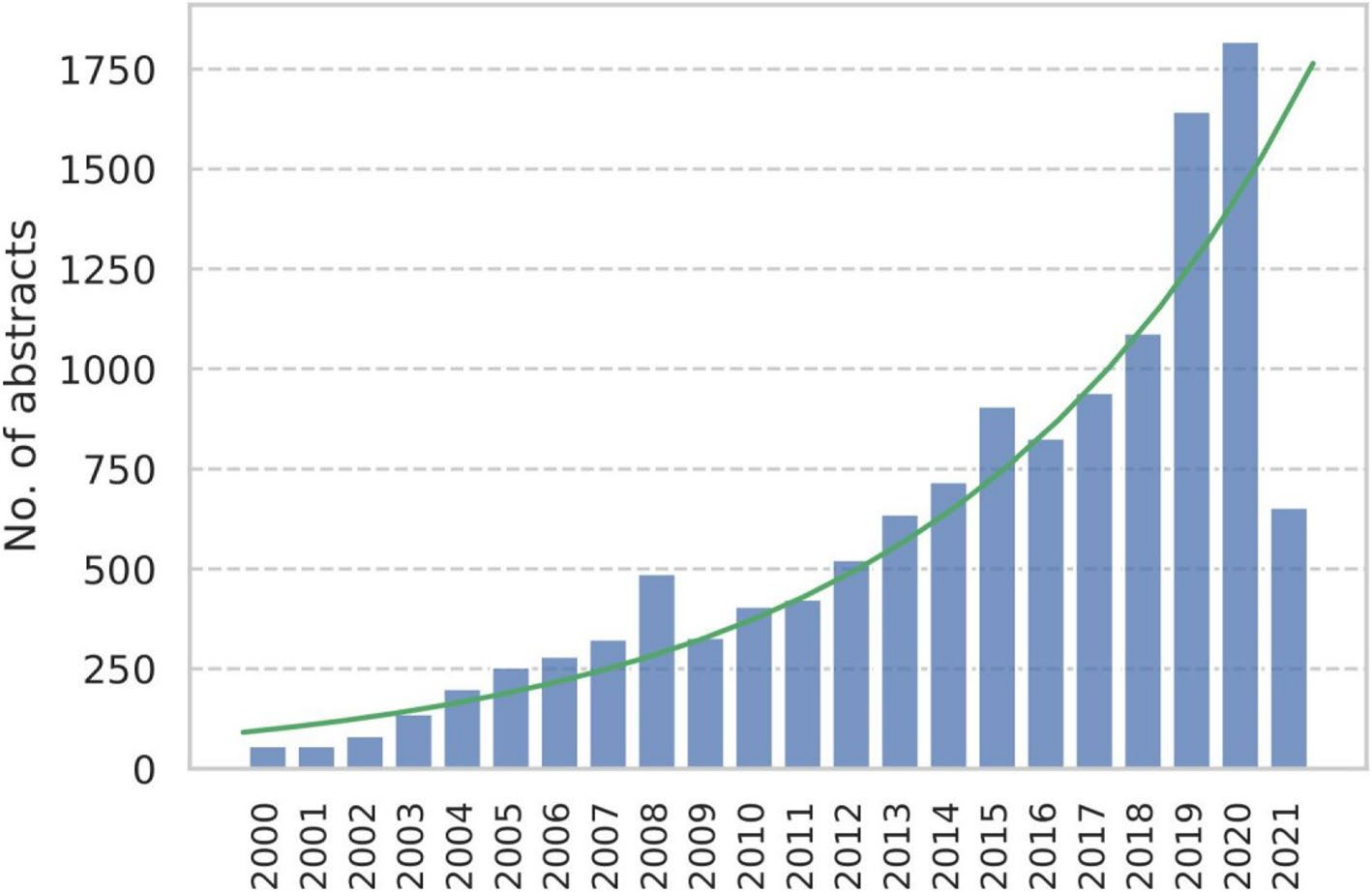
Trend analysis of 12,817 abstracts showing the overall trend of the growth and the number of articles per year

Based on the evaluation, the trained models classified abstracts accurately into their text source, context, and primary research field (disease area) by 78.5%, 77.3%, and 87.6%, respectively. Accordingly, the trained models’ micro f1-scores for classifying abstracts into their text source, context of use, and primary research field were 77.35%, 76.24%, and 85.64%, respectively. We retrieved 22,294 English abstracts from the database. There were 12,817 references left after removing 8815 duplicates, 500 articles without abstracts, 32 errata, 31 commentaries, 31 editorials, and 68 veterinary-related abstracts. The selected analyses were based on 12,161 abstracts, excluding those published in 2021. Figure 2 illustrates the abstract selection process for creating the final abstract collection. NLP publications have increased logarithmically since 2000, as shown in Fig. 3.

**Fig. 4:**
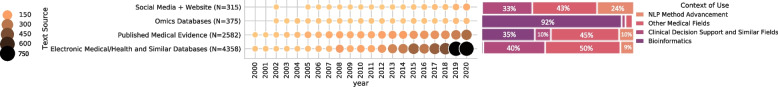
Proportion and growth of four selected subgroups of text source since the year 2000 and percentage of selected subgroups of the “context of use” of the total for the subgroups of text source

The Additional file 2 conveys the total number of abstracts retrieved for each subgroup. Table 2 shows the AAGR and average growth slope (coefficient) with a 95% confidence interval. It also displays the adjusted R2 of the regression model with and without a squared term for the publication year. The AAGR was 20.99%, with an average increase of 72 (95% *CI*: 56.80–78.30) publications per year. According to the adjusted R2 of 83%, the publication number is strongly affected by time. After adding a squared term for publication year, the indicator increased to 93%, indicating logarithmic growth. In all types of NLP text sources, electronic medical or health records or similar electronic clinical notes accounted for the highest percentage (57.12%). The addition of published articles and other sources of medical evidence accounted for 33.84% of all NLP text sources. Social media, including websites and databases with omics data (e.g., genomics), accounted for less than 10% of all NLP text sources (Table 2). Figure 4 displays the relative proportions and growth trends of four specific subgroups of text sources since the year 2000. Additionally, it presents the percentage representation of these chosen subgroups within the total for the “context of use” of the text sources. Despite comprising only 4.91% of publications, the so-called omics text data exhibited the fastest growth (*AAGR* = 65.08%) among all other text sources.

**Table 2:** Linear regression of the subgroups of each class

	Average annual growth rate	Proportion	Coefficient for the year of publication (95% *CI*)	*p*-value	Adjusted *R*^2^	Adjusted *R*^2^ (model with year squared)
All	20.99%	100%	72.01 (56.8–78.3)	< 0.001	0.83	0.93
Primary research fields						
Certain infectious or parasitic diseases	42.84%	9.84%	3.46 (1.92–4.90)	< 0.001	0.514	0.697
Mental, Behavioral, or Neurodevelopmental disorders	39.28%	16.27%	6.23 (4.28–8.19)	< 0.001	0.686	0.932
Neoplasm	42.41%	27.66%	9.8377 (7.498–12.18)	< 0.001	0.793	0.955
Diseases of the Circulatory System	49.04%	11.27%	3.78 (2.918–4.648)	< 0.001	0.805	0.912
Text sources						
Electronic Medical/Health and Similar databases	25.41%	57.12%	30.87 (23.33–38.41)	< 0.001	0.784	0.931
Published Medical Evidence	31.52%	33.84%	13.07 (11.247–14.9)	< 0.001	0.918	0.935
Social media + Website	51.03%	4.13%	3.133 (1.86–4.41)	< 0.001	0.56	0.874
Omics databases	65.08%	4.91%	1.66 (1.07–2.25)	< 0.001	0.627	0.637
Context of use						
Bioinformatics	69.65%	15.66%	5.26 (4.23–6.29)	< 0.001	0.849	0.853
Clinical Decision Support and Similar fields	32.12%	25.45%	19.25 (13.42–25.09)	< 0.001	0.7	0.94
NLP Method Advancement	31.76%	10.47%	7.09 (4.83–9.35)	< 0.001	0.678	0.896
Other Medical Fields	19.44%	48.42%	21.72 (17.486–25.96)	< 0.001	0.851	0.888

*NLP*; natural language processing

**Fig. 5:**
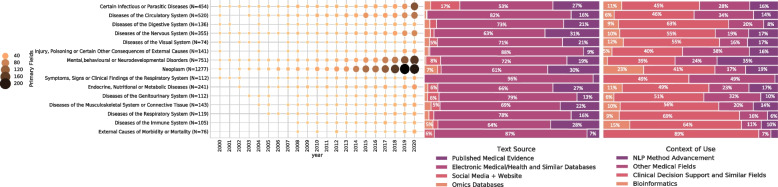
Proportion and growth of the most prevalent primary research fields since the year 2000 and the percentage of selected subgroups of the “context of use” and the “text source” of the total for the subgroups of the primary research field

**Fig. 6:**
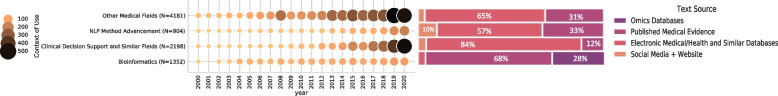
Proportion and growth of the “context of use” since the year 2000 and the percentage of selected subgroups of the “text source” of the total for the subgroups of the “context of use”

Changes in the dominant primary research fields since 2000, along with the expansion rates, as well as the distribution percentages for specific subcategories within “context of use” and “text source,” are illustrated in Fig. 5. Four medical fields accounted for slightly over 65% of all the research NLP researchers conducted and published (neoplasms, mental conditions, infectious diseases, and circulatory diseases). Neoplasms topped this list. The growth rates of all these medical fields were comparable (Table 2 and Fig. 5). NLP methods for clinical decision support were the most notable identifiable application among different aims (called “context of use”) of NLP studies, accounting for 25.45% of all publications. In contrast, bioinformatics-related discoveries showed the highest growth (*AAGR* = 69.65%) among all medical NLP applications, in line with the highest growth of omics databases. Among the subgroups under “context of use,” the majority belonged to “other medical fields,” which included a wide range of medical applications. Changes in the “context of use” since the year 2000, including its proportion and growth, along with the percentage representation of specific subgroups within the “text source,” are depicted in Fig. 6 (Table 2 and Fig. 6).

According to Fig. 5, clinical decision support applications and electronic medical/health records had the highest proportion of context of use and text source for each subgroup of primary research fields. The proportion of text source and context of use subtypes varied significantly across medical fields. For instance, published papers on NLP method advancement accounted for the highest percentage (35%) of ICD-11 codes for mental, behavioral, and neurodevelopmental disorders. Similarly, social media was used more frequently (17%) in certain infectious or parasitic diseases than in any diseases designated by ICD-11 codes.

## Discussion

Yu Zhu et al. [13] used output-modified BioBERT pre-trained with PubMed and PMC and obtained an f-score of 80.9, like ours. Elangovan et al. [11] found a lower f-score in a similar study. The other two systematic reviews observed a similar upward trend in using NLP in various medical fields over the last two decades [14, 15]. In 2000, medical NLP publications began to appear prominently in peer-reviewed journals. This study shows BioBERT can spot an expected result reported in previous studies.

We were particularly interested in the type of text sources used in medial NLP, the type of medical conditions studied, and the motivation behind performing NLP. Three published bibliographic studies shared some features with ours. Using PubMed data, Chen et al. examined 1405 papers over 10 years (2007–2016) and reported country-region, author-affiliation, and thematic NLP research areas [14]. Using PubMed data from 1999 to 2018, Wang et al. identified 3498 publications. Additionally, country-regions, author affiliations, disease areas, and text sources were reported [15]. Similar to Wang [15] and Chen [14], Chen et al. [17] used NLP methods to explore a similar set of variables; however, the authors focused only on NLP-enhanced clinical trial research. PubMed, Web of Science, and Scopus were searched for 451 published articles from 2001 to 2018. We selected 12,817 peer-reviewed citations using a different approach than typical bibliographic methods. We systematically scanned four chief article datasets and manually classified citations based on three variables: primary research fields, text source used, and motivation for NLP (context of use). In addition, we used BioBERT of Google as a preferred NLP method to assign subgroups to our variables.

Unlike typical bibliometric research, we were not interested in regional or institutional distributions, typical features of bibliometric research. Instead, we explored the hows and whys of medical NLP research over the past two decades.

According to our results, annual medical NLP publications grew by roughly 21% between 2000 and 2020 on average, similar to the nearly 18% growth. Chen et al. reported between 2007 and 2016 [14]. According to Wang et al. [15] and Chen et al. [17], medical NLP publications increased rapidly between 1999 and 2017. The logarithmic progression of the citations in our study can partly be explained by the annual increase of over 65% in NLP studies using omics datasets. Nearly 27% of all NLP research was conducted on neoplasms, mental conditions, infectious diseases, and circulatory diseases. Similarly, Wang et al. retrieved around 25% of their citations from neoplasms [15]. Previous authors have not explained why medical NLP citations are unequally high in cancer and a limited number of other fields, like mental health. The same is valid for why particular medical conditions are at the center of medical community researchers, while EHR (electronic health records) or EMR (electronic medical records) massive data must be equally available for all medical conditions proportional to their prevalence. In the case of cancers and infectious lung disease, however, unstructured text may convey more information because of pathology or radiology reports. We can potentially apply medical NLP to broader clinical and research settings by studying the systematic differences across medical conditions from an NLP standpoint.

There are strengths and weaknesses to our approach. We began by categorizing medical conditions hierarchically using a systemic strategy. To identify primary research fields, we used ICD-11’s top-level taxonomy. In the future, if NLP studies follow the same procedure, the findings will remain comparable. We chose the BioBERT model from various pre-trained language models, including ClinicalBERT and BlueBERT. BioBERT can train with 4.5 billion biomedical words from PubMed abstracts and 13.5 billion words from PMC full-text articles. Compared to similar BERT models, NLP researchers are more involved with BioBERT. Hence, we recommend comparing the performance of various BERT models before selecting a model if an NLP specialist is not confident enough to choose the proper model. Finally, we publish the method for developing the analysis database (NLP corpus) based on medical systematic review guidelines. Future research can use this approach to confirm whether NLP can replace systematic literature reviews.

A potential shortcoming of our study was the idiosyncratic nature of the initial classification used for training the machine. Using our experience with observational datasets, such as electronic clinical notes and NLP applications, to analyze unstructured clinical data, we began building the initial subgroups. Nevertheless, to mitigate the risk of bias, we dissected the published studies cumulatively until more studies could not update the evolving classification. Our models’ estimated classification accuracy may have been adequate because of this strategy. The model can be fine-tuned based on more annotated articles, the hyper-parameters can be tuned more thoroughly, and multi-task learning can be explored instead of training separate models for each task. Additionally, the accuracy may improve further after the training dataset is expanded. Finally, we only included abstracts written in English. Results and conclusions may be influenced if relevant research published in languages other than English is excluded.

## Conclusions

This study aimed to evaluate the performance of BioBERT as a tool to substitute manual abstract review using a language model. BioBERT is an acceptable method for abstract selection for systematic literature searches since it uses a uniform and human-independent algorithm that reduces the time required for manual abstract selection and increases inter-study reliability.

### Supplementary Information

**Additional file 1**: **Appendix 1**. The three tables (a, b, and c) show the absolute number of abstracts retrieved between 2000 and 2020 (inclusive) for each of the three classes studied.

**Additional file 2**: **Appendix 2**. The search strategy used to received abstracts from four databases.

## Data Availability

Data sharing is not applicable, but the authors are happy to share the abstracts publicly if that helps the reviewers or readers.

## Abbreviations

NLP: Natural language processing
AAGR: Average annual growth rate
EHR: Electronic health records
EMR: Electronic medical records
ICD: International Classification of Diseases
BERT: Bidirectional Encoder Representations from Transformers
