# Supplementary material for: Natural language processing (NLP) to facilitate abstract review in medical research: the application of BioBERT to exploring the 20-year use of NLP in medical research
Source: Syst Rev. 2024 Apr 15;13:107. doi: 10.1186/s13643-024-02470-y (PMC11020656; doi:10.1186/s13643-024-02470-y)
Supplement: Supplementary file 1 — Additional file 1: Appendix 1. The three tables (a, b, and c) show the absolute number of abstracts retrieved between 2000 and 2020 (inclusive) for each of the three classes studied. [file 13643_2024_2470_MOESM1_ESM.docx]

| 1. Primary medical fields according to ICD-11 | No of abstracts |
| --- | --- |
| Not Related (NR) | 7374 |
| neoplasm | 1277 |
| mental, behavioural or neurodevelopmental disorders | 751 |
| diseases of the circulatory system | 520 |
| certain infectious or parasitic diseases | 454 |
| diseases of the nervous system | 355 |
| endocrine, nutritional or metabolic diseases | 241 |
| diseases of the musculoskeletal system or connective tissue | 143 |
| injury, poisoning or certain other consequences of external causes | 141 |
| diseases of the digestive system | 136 |
| diseases of the respiratory system | 119 |
| diseases of the genitourinary system | 112 |
| symptoms, signs or clinical findings of the respiratory system | 112 |
| diseases of the immune system | 105 |
| external causes of morbidity or mortality | 76 |
| diseases of the visual system | 74 |
| diseases of the skin | 44 |
| problems associated with harmful or traumatic events | 37 |
| pregnancy, childbirth or the puerperium | 29 |
| factors influencing health status or contact with health services | 18 |
| supplementary section for functioning assessment | 16 |
| neuromusculoskeletal and movement-related functions | 10 |
| symptoms, signs or clinical findings of the digestive system or abdomen | 9 |
| diseases of the urinary system | 7 |
| symptoms, signs or clinical findings, not elsewhere classified | 1 |
| Total | 12161 |

Appendix 1. The three tables ( a, b, and c) show the absolute number of abstracts retrieved between 2000 and 2020 (inclusive) for each of the three classes studied.

| 1. Context of use | No of abstracts |
| --- | --- |
| waste basket collection | 3526 |
| Other Medical Fields | 4181 |
| Clinical Decision Support and Similar Fields | 2198 |
| Bioinformatics | 1352 |
| NLP Method Advancement | 904 |
| Total | 12161 |

| 1. Text-source | No of abstracts |  |
| --- | --- | --- |
| Electronic Medical/Health and Similar Databases | 4358 |  |
| NR | 3977 |  |
| Published Medical Evidence | 2582 |  |
| Miscellaneous | 451 |  |
| Omics Databases | 375 |  |
| Social Media + Website | 315 |  |
| ND | 98 |  |
| Questionnaire | 4 |  |
| Interview | 1 |  |
| Total | 12161 |  |
