# Supplementary material for: Natural language processing (NLP) to facilitate abstract review in medical research: the application of BioBERT to exploring the 20-year use of NLP in medical research
Source: Syst Rev. 2024 Apr 15;13:107. doi: 10.1186/s13643-024-02470-y (PMC11020656; doi:10.1186/s13643-024-02470-y)
Supplement: Supplementary file 2 — Additional file 2: Appendix 2. The search strategy used to received abstracts from four databases. [file 13643_2024_2470_MOESM2_ESM.docx]

Appendix 2. The search strategy used to received abstracts from four databases

| PubMed Query – April 28, 2021 | Items found |
| --- | --- |
| "natural language processing"[Title/Abstract] OR "natural language processing"[MeSH Terms] OR "text mining"[Title/Abstract] |  |
| Filters applied:  Case Reports, Classical Article, Clinical Conference, Clinical Study, Clinical Trial, Clinical Trial Protocol, Clinical Trial, Phase I, Clinical Trial, Phase II, Clinical Trial, Phase III, Clinical Trial, Phase IV, Validation Study, Clinical Trial, Veterinary, Comparative Study, Controlled Clinical Trial, Corrected and Republished Article, Dataset, Duplicate Publication, English Abstract, Evaluation Study, Guideline, Interactive Tutorial, Introductory Journal Article, Journal Article, Meta-Analysis, Multicenter Study, Observational Study, Pragmatic Clinical Trial, Preprint, Randomized Controlled Trial, Retracted Publication, Review, Scientific Integrity Review, Systematic Review, Twin Study.  2000-2021 |  |
|  | 8,773 |
| EMBASE Query – April 28,2021 | Items found |
| ('natural language processing':ti,ab,kw OR 'natural language processing'/exp OR 'text mining':ti,ab,kw) AND [2000-2021]/py |  |
| AND ('article'/it OR 'article in press'/it OR 'conference abstract'/it OR 'conference paper'/it OR 'conference review'/it OR 'data papers'/it OR 'review'/it OR 'short survey'/it) |  |
|  | 9,642 |

| CINAHL Query – April 28, 2021 | Items found |
| --- | --- |
| TI ( natural language processing or nlp ) OR AB ( natural language processing or nlp ) OR TI text mining OR AB text mining |  |
| Filters applied:  ACADEMIC JOURNAL  2000-2021 |  |
|  | 1951 |

| PsycINFO– April 28, 2021 | Items found |
| --- | --- |
| MA ( natural language processing or NLP ) OR TI ( natural language processing or NLP ) OR AB ( natural language processing or NLP ) OR TI text mining OR AB text mining |  |
| Filters applied:  ACADEMIC JOURNAL  2000-2021 |  |
|  | 1928 |
